# Comparative effectiveness of branded vs. generic versions of antihypertensive, lipid-lowering and hypoglycemic substances: a population-wide cohort study

**DOI:** 10.1038/s41598-020-62318-y

**Published:** 2020-04-06

**Authors:** Yuxi Tian, Berthold Reichardt, Daniela Dunkler, Milan Hronsky, Wolfgang C. Winkelmayer, Anna Bucsics, Susanne Strohmaier, Georg Heinze

**Affiliations:** 1 Department of Biomathematics, University of California Los Angeles, Los Angeles, CA USA; 2 Österreichische Gesundheitskasse, Eisenstadt, Austria; 3 Center for Medical Statistics, Informatics, and Intelligent Systems, Section for Clinical Biometrics, Medical University of Vienna, Vienna, Austria; 4 Section of Nephrology, Baylor College of Medicine, Houston, TX USA; 5 MoCA, Mechanism of Coordinated Access to Orphan Medicinal Products, Austria Vienna,

**Keywords:** Interventional cardiology, Health services

## Abstract

Generic medications offer substantial potential cost savings to health systems compared to their branded counterparts. In Europe and the US, they are only approved if they are bioequivalent to the respective originator product. Nevertheless, the lack of clinical outcomes is sometimes used as the reason for hesitancy in prescribing generics. We performed an observational retrospective study on 17 branded vs. generic pharmaceutical substances for the treatment of hypertension/heart failure, hyperlipidemia, and diabetes mellitus in a dataset of 9,413,620 insured persons, representing nearly the full population of Austria, from 2007 to 2012. We compared generic vs. branded medications using hazard ratios for all-cause death and major adverse cardiac and cardiovascular events (MACCE) as outcomes of interest. Using patient demographics, health characteristics from hospitalization records, and pharmacy records as covariates, we controlled for confounding in Cox models through inverse probability of treatment weighting (IPTW) using high-dimensional propensity scores. We observed that the unadjusted hazard ratios strongly favor generic drugs for all three pooled treatment indications (hypertension/heart failure, hyperlipidemia, diabetes mellitus), but were attenuated towards unity with increasingly larger covariate sets used for confounding control. We found that after IPTW adjustment the generic formulation was associated with significantly fewer deaths in 10 of 17 investigated drugs, and with fewer MACCE in 11 of 17 investigated drugs. This result favoring generic drugs was also present in a number of sub-analyses based on gender, prior disease status, and treatment discontinuation. E-value sensitivity analyses suggested that only strong unmeasured confounding could fully explain away the observed results. In conclusion, generic medications were at least similar, and in some cases superior, to their branded counterparts regarding mortality and major cardiovascular events.

## Introduction

Generic medications offer potential for substantial health care cost savings compared to their branded drug counterparts^1–3^, but their adoption is hindered by doubts among physicians and patients regarding their efficacy and safety^4–8^. Some of the concerns arise from a lack of knowledge or misinformation regarding the concept of bioequivalence and/or from marketing efforts of branded drug manufacturers. Frequently, the argument is that while pharmaceutical companies need to conduct extensive clinical trials to bring an innovator branded drug to market, they are required only to demonstrate biologic equivalence for new generic drugs, and not equivalence in clinical outcomes^9^. However, randomized controlled trials of generic drugs vs originators are rarely feasible or required by regulators, unless bioequivalence cannot be shown with pharmacokinetic studies, e.g. for drugs not administered systemically (https://www.ema.europa.eu/en/human-regulatory/marketing-authorisation/generic-hybrid-medicines, accessed 07/29/2019). As a result, randomized trials comparing generic to branded drugs feature relatively small sample sizes that are sufficient to show bioequivalence, but are by their very nature not powered to find significant differences in clinical efficacy^10–12^.

In the absence of randomized trials, retrospective data are a crucial resource to collect clinical data on generic drugs^13^. Observational studies conducted using longitudinal health databases that contain millions of patient records could discern clinically meaningful differences between branded and generic drugs. However, these studies require careful control for potential confounding that plagues all observational research. Previous studies in this field have highlighted the need to control not only for patient medical history, but also additional factors such as socioeconomic status^14^ and medication adherence^15^.

There has been extensive observational research on antiepileptic drugs, including several narrow therapeutic index drugs that are particularly concerning for introducing generic alternatives^16–19^. There are relatively fewer studies on generic medications for chronic metabolic diseases such as hypertension or heart failure, hyperlipidemia, and diabetes mellitus that offer significant opportunities for cost savings owing to their widespread use^20^. In this study, we compared death and cardiovascular outcomes between branded and generic formulations of 17 antihypertensive, cholesterol-lowering, and oral hypoglycemic drugs using national pharmacy and hospitalization data representing nearly all insured persons in Austria.

## Methods

### Study population and data

We analyzed all filled prescriptions that were submitted for reimbursement to 13 Austrian social security institutions, including all nine provincial sickness funds as well as four nationwide institutions (federal employees, farmers, independent business owners, and railroad and mining employees). In total, these institutions cover 98.5% of all insured persons in Austria. Prescription data were available for 9,413,620 insured persons from 2007 to 2012, and each record contained a pseudonymized unique patient identifier, volume (number of packages), package size (number of units per package), strength (dose per unit), the pharmacy article identifier of the dispensed drug, and patient co-payment waiver status (yes or no). In addition, linked through the pseudonymized patient identifier we obtained patient birth months, sex, date of deregistration from the social security institution (if applicable), date of death (if applicable), and all hospitalizations in the study period including admission date, length of stay, and discharge diagnoses. These data have been utilized and described in previous studies comparing generic and branded drug costs^3^ and investigating double medication rates^21^.

### Investigated drug classes

For each of the investigated chronic diseases (hypertension or heart failure, hyperlipidemia, and diabetes mellitus) we compiled a list of therapeutic substances in terms of the World Health Organization (WHO)’s fifth-level (seven digit) Anatomical Therapeutic Chemical (ATC) code^22^ as previously reported^3^. From this list, we selected the 17 substances with the highest potential monetary savings that could be achieved by generic substitution^3^, and for which generic and branded versions were simultaneously available in the same combination of package size and strength. This list comprised twelve single substances or substance combinations for hypertension or heart failure treatment (metoprolol, bisoprolol, nebivolol, carvedilol, amlodipine, enalapril, lisinopril, ramipril, enalapril and diuretics, lisinopril and diuretics, ramipril and diuretics, losartan and diuretics), two lipid-lowering substances (simvastatin, fluvastatin), and three oral hypoglycemic substances (metformin, gliclazide, repaglinide). A database supplied by the Austrian Agency for Health and Food Safety (AGES) provided information on the branded versus generic status of each pharmacy article. For each drug class we defined a start date of the study period as the date at which a generic version of the drug was first reimbursed in our database. Thus, data from branded medicines were only considered starting with the month when a generic was also available. Only specific package size/strength combinations for which both generic and branded products were available were considered.

### Patient inclusion

**Figure 1:**
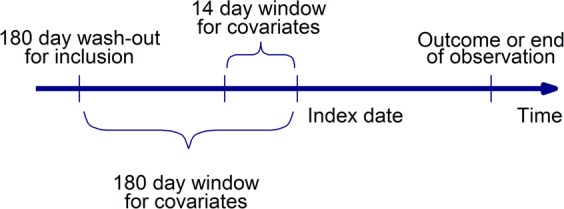
Data harvesting for the study. The inclusion date was the date of the first prescription or hospital admission of a patient in the data base. The index date was the date of first prescription of a study medication after a wash-out period of at least 6 months with no prescriptions of medicines of the same ATC code. All patients with an index date occurring at least 6 months after the inclusion date were included. Covariates (hospital discharge diagnoses, prescriptions, hospitalization days) were harvested during the 6 months preceding the index date. The patients were followed-up in the data base until an outcome event (death, MACCE), until deregistration from the insurer or until 31 December 2012, whichever occurred earlier.

Patients were included when they filled a new prescription of any of the investigated substances. This index date was used to determine subsequent study outcomes and ascertain preceding covariates. Only patients who were at least 18 years old at the index date were considered in the analysis. A wash-out period of at least 180 days with no prescription of the substance of interest was required to define a “new prescription” and to harvest covariates, and therefore, patients were excluded if the wash-out period was not fully covered by our database (Fig. 1). If a patient was simultaneously eligible for inclusion for multiple substances, we randomly selected one substance for that patient and hence included the patient only once in our study.

### Ascertainment of study outcomes

As primary outcomes, we considered time to all-cause death and time to major cardiac or cerebrovascular events (MACCE). We defined MACCE as any myocardial infarction, stroke, transient ischemic attack, or all-cause death. Ascertainment of MACCE was based on the following ICD10 codes recorded in hospital discharge diagnoses: I20, I21, I60, I61, I62, I63, I64, I65, I66, I69, G45. The starting point of these analyses was the time of index prescription. If patients had no events recorded in the database, we censored them at the date of their last observation date or at the date of deregistration from insurance, whichever occurred first. Treatment discontinuation was defined as no refill of initial type of prescription (branded or generic) within 180 days, conditional on survival and follow-up of at least 180 days.

### Ascertainment of covariates

The following covariates were harvested at the date of index prescription: age at prescription, sex, insurer, copayment waiver status, specialty of prescriber (general practitioner, internal medicine specialist, hospital, other), and year of prescription. Within the time period of 14 days preceding the index prescription, we extracted binary variables indicating whether any hospitalization ended in that period, whether a “long” hospitalization (duration of more than 14 days) ended in that period, an indicator for each discharge diagnosis recorded, and indicators for each drug class prescribed (ATC2 level). The same set of variables was also extracted for the time period of 180 to 14 days preceding the index prescription.

### Statistical analyses

A high-dimensional propensity score as outlined in Schneeweiss *et al*.^23^ describing the probability of receiving branded versus generic medication was fitted to calculate inverse probability of received treatment weights (IPTW). Specifically, we included the main descriptors (age, a quadratic age term (age/100)^2^, sex, any hospitalization in 180 day and 14 day windows, any discharge diagnosis indicating myocardial infarction or cerebrovascular events), the 200 variables with the highest potential to correct for bias^23,24^, two-way interactions among the main descriptors, and interactions between these main descriptors and diagnoses and prescriptions. We applied the least angle shrinkage and selection operator (lasso) to regularize and perform model selection among the interaction terms^25^. We used IPTW to equalize differences in the characteristics between patients receiving branded drugs and patients receiving generic drugs as index prescription. We evaluated success of propensity score weighting by comparing standardized mean differences in all covariates before and after weighting.

Kaplan-Meier curves were used to describe time to death and time to MACCE. Ninety-five percent confidence intervals (CI) for incidence rates were computed using a Poisson distribution. Cox proportional hazards regression models were used to estimate unadjusted and adjusted hazard ratios and 95% confidence intervals (95%CI) for all-cause death and MACCE, and logistic regression was used to compute unadjusted and adjusted relative risks and 95%CI for treatment discontinuation.

For each substance, we estimated hazard ratios for mortality as well for MACCE with the following adjustment levels:

- unadjusted,
- minimally adjusted (adjusted for age, (age/100)^2^, sex, and copayment waiver status),
- adjusted by an extended set of covariates (minimal adjustment set plus calendar year of index prescription, specialty of prescriber, previous hospitalizations, recent MI or cerebrovascular events, any diagnosis in group of endocrine, nutritional or metabolic diseases (ICD10 code E) or in group of diseases of circulatory system (ICD10 code I), and any previous use of antihypertensive, lipid-lowering or hypoglycemic drugs),
- fully adjusted by IPTW weighting (aHR). IPTW-adjusted models were also subgrouped by sex, by age (<= or >70 years), by any history of cardiovascular or diabetes disease (CVDD) as evidenced by previous diagnosis codes or relevant drug prescriptions, and by diabetes treatment status (no diabetes vs. oral hypoglycemic drugs prescribed but no insulin vs. insulin prescribed).

As a sensitivity analysis for potential unmeasured confounding we calculated E-values for point estimates and confidence limits according to VanderWeele and Ding^26^. E-values quantify the minimum strength of an association between a hypothetical unmeasured confounder and both treatment and outcome that could account for the observed treatment effect after controlling for measured covariates. We investigated time-dependency of treatment effect estimates by estimating adjusted hazard ratios during the first six months (censoring later events) and during the period after six months (conditional on surviving six months). These analyses were accompanied by interaction testing.

To investigate the role of treatment discontinuation, three additional models were fitted for each outcome (IPTW adjusted):

- a landmark model, conditional on survival of 6 months, including treatment discontinuation before 6 months as covariate,
- a subgroup landmark model with landmark set at 6 months including only patients who continued treatment within 6 months from initial prescription,
- and a subgroup landmark model (6 months) including only patients who discontinued treatment within 6 months from initial prescription.

All hazard ratios were estimated separately for each substance and were then pooled across all substances of the same indication (antihypertensive drugs, lipid-lowering drugs, hypoglycemic drugs) using random-effects meta-analysis. If weighted models were estimated, then a robust covariance matrix was used. All models were stratified for package size/strength combination at initial prescription.

Data were analyzed using PostGreSQL^27^ and R^28^.

### Ethics, data protection and data availability

The protocol of the study was created in compliance with the Guidelines for Good Pharmacoepidemiology Practices^29^. According to the Austrian Federal Act concerning the Protection of Personal Data (‘Datenschutzgesetz’, DSG) the study was exempted from the need to obtain informed consent from the participants as the research data base which was provided by the Main Association of the Austrian Social Security Institutions was already irreversibly pseudonymized and the identities of the participants could not be established. As this was a retrospective study, participation in the study did not alter any risks of the participants. The study protocol and the exempt from the need to obtain informed consent was approved by the Ethics Committee of the Medical University of Vienna (ECS 1533/2013). The data that support the findings of this study are available from the Main Association of the Austrian Social Security Institutions but restrictions apply to the availability of these data, which were used under license for the current study, and so are not publicly available. Data are however available from the authors upon reasonable request and with permission of the Main Association of the Austrian Social Security Institutions.

## Results

### Patients

**Figure 2:**
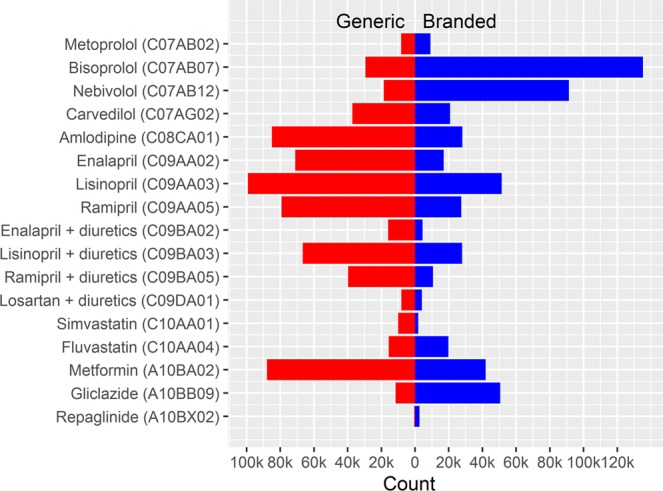
Patient counts (1k = 1,000) for each substance evaluated.

During the study period from 2007 to 2012, 986,149; 47,359; and 201,038 patients with index prescriptions for antihypertensive, lipid-lowering and hypoglycemic drugs were included, respectively, with follow-up totaling to 1,920,544; 93,952; and 383,460 patient years. Figure 2 shows patient counts for each evaluated substance, grouped by branded or generic medicines. Characteristics of patients at their first index prescription are displayed in Table 1. In general, patients receiving branded medicines were older, more often had recent (within past 14 days) or previous (within past 180 days) hospitalizations, more often had used antihypertensive, lipid-lowering and hypoglycemic drugs before and more often received their index prescriptions from hospitals compared to patients treated with generic medicines. For lipid-lowering and hypoglycemic drugs, we also observed that men received branded medicines more often than women.

**Table 1:** Characteristics of patients at first index prescription for antihypertensive, lipid-lowering or hypoglycemic treatment.

Variable	Branded anti-hypertensive (N = 427,641)	Generic anti-hypertensive (N = 558,508)	Branded lipid-lowering (N = 21,665)	Generic lipid-lowering (N = 25,694)	Branded hypo-glycemic (N = 101,045)	Generic hypo-glycemic (N = 99,993)
Age (years), mean (standard deviation)	64.5 (15.4)	63.3 (14.5)	63.0 (12.8)	62.4 (12.5)	65.0 (13.9)	62.7 (13.5)
Sex: female	54.3%	54.5%	50.3%	54.3%	49.5%	50.2%
Copayment waiver	34.2%	29.9%	30.6%	26.3%	41.1%	38.3%
Hospitalization (ending in last 180 days)	30.0%	19.9%	23.0%	18.1%	26.2%	18.7%
Hospitalization >14 days (ending in last 14 days)	7.3%	2.5%	3.7%	2.0%	5.7%	2.0%
Hospitalization >14 days (ending in last 180 days)	10.8%	5.4%	6.9%	4.6%	9.1%	4.9%
**Index year:**						
2007	0.7%	0.6%	0.1%	0.4%	1.0%	0.7%
2008	10.1%	9.1%	1.4%	4.5%	11.2%	8.2%
2009	22.4%	22.0%	33.3%	16.9%	22.4%	18.4%
2010	27.0%	24.9%	28.1%	35.5%	25.6%	25.7%
2011	20.6%	23.3%	22.1%	25.4%	22.2%	25.4%
2012	19.2%	20.1%	15.0%	17.3%	17.6%	21.5%
**Specialty of prescriber:**						
General practitioner	67.7%	78.5%	68.9%	75.4%	77.5%	81.7%
Internal medicine specialist	11.6%	13.3%	12.8%	15.5%	9.7%	10.4%
Hospital	9.8%	3.7%	10.2%	3.7%	5.7%	2.8%
Other	10.8%	4.6%	8.1%	5.4%	7.1%	5.2%
Recent myo-cardial infarction	2.5%	0.6%	3.8%	1.1%	0.9%	0.3%
Recent cerebro-vascular event	2.3%	1.1%	2.3%	1.6%	1.6%	0.6%
Any diagnosis in group of endocrine, nutritional or metabolic diseases or in group of diseases of circulatory system	33.0%	16.6%	25.3%	15.5%	28.0%	15.5%
Previous use of antihypertensive, lipid-lowering or hypoglycemic medicines	67.5%	64.1%	73.0%	69.0%	81.3%	76.4%
Previous use of injectable insulins	1.9%	1.4%	2.0%	1.4%	3.2%	2.5%
Previous use of oral hypoglycemic drugs	12.7%	11.5%	14.6%	13.0%	39.2%	21.6%

Propensity models achieved concordance indices in the range of 0.639 (enalapril, C09AA02) to 0.854 (losartan and diuretics, C09DA01). After IPTW weighting, maximum standardized mean differences across all high-dimensional propensity score covariates were below 10% for all substances, except for repaglinide, A10BX02 (17.3%) and bisoprolol, C07AB07 (12.4%, Supplementary Table 1). The means of the standardized mean differences across all covariates were always <3%, and were <1% for 15 of the 17 studied substances.

### Anti-hypertensives: primary time-to-event outcomes

**Figure 3:**
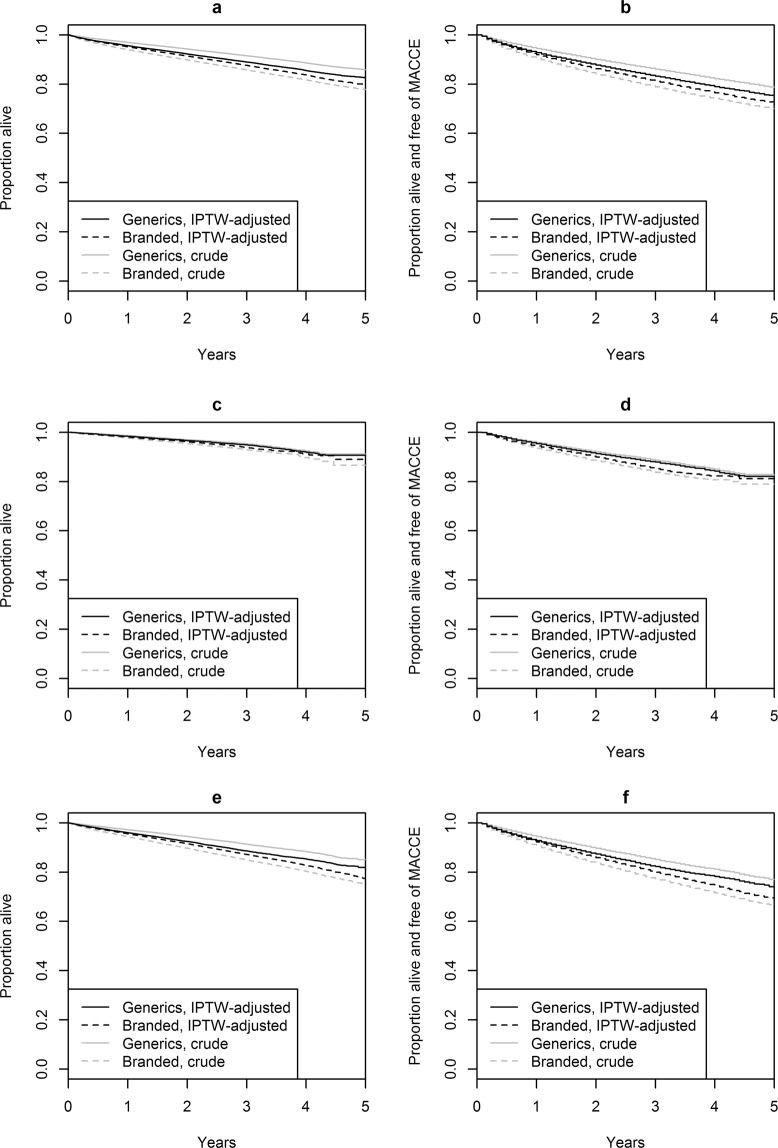
Survival curves and curves of cumulative MACCE-free survival. (**a**) Overall survival for patients with index prescriptions for antihypertensive drugs. (**b**) MACCE-free survival for patients with index prescriptions for antihypertensive drugs. (**c**) Overall survival for patients with index prescriptions for lipid-lowering drugs. (**d**) MACCE-free survival for patients with index prescriptions for lipid-lowering drugs. (**e**) Overall survival for patients with index prescriptions for hypoglycemic drugs. (**f**) MACCE-free survival for patients with index prescriptions for hypoglycemic drugs.

Across all 12 antihypertensive substances, 53.8 (95% CI; 53.3, 54.3) deaths per 1000 patient-years were observed for branded medicines, while the corresponding figure was 30.2 (95% CI; 29.9, 30.5) for generic medicines. After IPTW adjustment, the estimated incidence rates were 45.8 (95% CI; 45.5, 46.1) deaths per 1000 patient years for branded medicines and 40.6 (95% CI; 40.4, 40.9) for generic medicines, Crude cumulative five-year survival rates in branded and generics users were 77.8% (95% CI; 77.3%, 78.4%) and 85.9% (95% CI; 85.5%, 86.2%), respectively, and the corresponding IPTW-adjusted rates were 79.8% (95% CI; 79.4%, 80.1%) and 82.7% (95% CI; 82.4%, 83.0%) (Fig. 3). The unadjusted pooled branded vs. generic hazard ratio (HR) of 1.75 (95% CI; 1.56, 1.98) was attenuated after IPTW adjustment to an aHR of 1.15 (95% CI; 1.06, 1.26), favoring generics. Table 2 illustrates the aHR resulting from different adjustments, demonstrating a continuously decreasing aHR with increasing covariate adjustment. Table 3 compares fully adjusted aHRs across different substances. Interestingly, while results for most substances favored of generics, the direction of association was reversed for bisoprolol (C07AB07) and nebivolol (C07AB12). Among all subgroup analyses conducted, we only found significant treatment effect modification with history of CVDD (interaction p < 0.001). In patients without CVDD history, the aHR was 1.47 (95%CI; 1.31, 1.64), while being only 1.10 (95%CI; 1.01, 1.20) in patients with CVDD history.

**Table 2:** Pooled IPTW-adjusted hazard ratios (HR) for all-cause mortality and major cardiac or cerebrovascular events (MACCE) comparing branded vs. generic medicines applying different levels of adjustment.

Indication	Adjustment variables	Mortality HR (95%CI) for branded vs. generic	MACCE HR (95%CI) for branded vs. generic
Antihypertensive drugs	No adjustment	1.75 (1.56, 1.98)	1.62 (1.47, 1.77)
	Age, sex, copayment waiver	1.52 (1.37, 1.69)	1.44 (1.33, 1.56)
	Extended set of covariates^*^	1.23 (1.13, 1.34)	1.18 (1.12, 1.25)
	**IPTW via high-dimensional propensity scores**	**1.15** (**1.06, 1.25)**	**1.13** (**1.07, 1.20)**
	E-value (lower 95% confidence limit)^**^	1.57 (1.31)	1.51 (1.34)
Lipid-lowering drugs	No adjustment	1.69 (1.09, 2.63)	1.49 (1.04, 2.12)
	Age, sex, copayment waiver	1.40 (0.84, 2.32)	1.32 (0.89, 1.96)
	Extended set of covariates^*^	1.33 (1.07, 1.64)	1.26 (1.17, 1.35)
	**IPTW via high-dimensional propensity scores**	**1.13** (**0.86, 1.47)**	**1.20** (**1.05, 1.38)**
	E-value (lower 95% confidence limit)^**^	1.51 (1)	1.69 (1.28)
Hypoglycemic drugs	No adjustment	1.43 (1.37, 1.49)	1.35 (1.31, 1.39)
	Age, sex, copayment waiver	1.32 (1.24, 1.40)	1.29 (1.26, 1.33)
	Extended set of covariates^*^	1.11 (1.01, 1.23)	1.10 (1.01, 1.18)
	**IPTW via high-dimensional propensity scores**	**1.09** (**0.93, 1.28)**	**1.11** (**1.03, 1.20)**
	E-value (lower 95% confidence limit)^**^	1.4 (1)	1.45 (1.21)

*Age, sex, copayment waiver status, calendar year, specialty of prescriber, previous hospitalizations, recent MI or cerebrovascular events, any diagnosis in group of endocrine, nutritional or metabolic diseases or in group of diseases of circulatory system, any previous use of antihypertensive, lipid-lowering or hypoglycemic drugs.

^**^E-values (VanderWeele and Ding, 2017) for IPTW adjusted point estimate and lower confidence limit.

**Table 3:** IPTW-adjusted hazard ratios (HR) and 95% confidence intervals (CI) of all-cause mortality and major cardiac or cerebrovascular events (MACCE) for individual substances.

*Indication* Substance	ATC code	N branded	N generics	Mortality: HR (95%CI) for branded vs. generic	MACCE: HR (95%CI) for branded vs. generic
*Antihypertensive drugs*					
Metropolol	C07AB02	9,185	8,202	1.13 (0.96, 1.32)	1.15 (1.01, 1.30)
Bisoprolol	C07AB07	135,208	29,442	0.84 (0.76, 0.92)	0.91 (0.85, 0.98)
Nebivolol	C07AB12	91,283	18,561	0.81 (0.68, 0.97)	0.98 (0.86, 1.11)
Carvedilol	C07AG02	20,837	37,181	1.19 (1.10, 1.28)	1.17 (1.10, 1.25)
Amlodipine	C08CA01	28,118	84,988	1.41 (1.35, 1.48)	1.28 (1.23, 1.33)
Enalapril	C09AA02	17,053	71,065	1.08 (1.02, 1.15)	1.06 (1.01, 1.11)
Lisinopril	C09AA03	51,443	99,145	1.15 (1.10, 1.20)	1.17 (1.13, 1.21)
Ramipril	C09AA05	27,388	79,301	1.32 (1.24, 1.41)	1.26 (1.20, 1.33)
Enalapril and diuretics	C09BA02	4,492	16,024	1.08 (0.96, 1.22)	1.01 (0.91, 1.12)
Lisinopril and diuretics	C09BA03	27,967	66,727	1.24 (1.16, 1.32)	1.19 (1.13, 1.25)
Ramipril and diuretics	C09BA05	10,643	39,711	1.25 (1.15, 1.37)	1.23 (1.15, 1.32)
Losartan and diuretics	C09DA01	4,024	8,161	1.64 (1.23, 2.20)	1.20 (0.98, 1.46)
*Lipid-lowering drugs*					
Simvastatin	C10AA01	1,862	10,079	0.97 (0.76, 1.42)	1.09 (0.87, 1.33)
Fluvastatin	C10AA04	19,803	15,615	1.28 (1.05, 1.56)	1.26 (1.14, 1.40)
*Hypoglycemic drugs*					
Metformin	A10BA02	41,889	87,929	1.21 (1.15, 1.26)	1.16 (1.11, 1.20)
Gliclazide	A10BB09	50,520	11,504	1.02 (0.94, 1.11)	1.08 (1.01, 1.16)
Repaglinide	A10BX02	2,636	560	0.91 (0.57, 1.45)	0.83 (0.57, 1.22)

In patients receiving branded medicines, we observed a rate of 83.6 (95% CI; 82.9, 84.2) major cardiac and cerebrovascular events (MACCE) per 1000 patient-years, compared to 51.3 (95% CI; 50.9, 51.8) in patients using generic medicines. The IPTW-adjusted MACCE incidence rates were 72.3 (95% CI; 72.0, 73.3) and 64.1 (95% CI; 63.8, 65.0). After IPTW adjustment, the hazard ratio for MACCE was 1.13 (95% CI; 1.07, 1.20). While most individual substances favored generic drugs, an opposite effect estimate was observed for bisoprolol (C07AB07) with aHR 0.91 (95% CI; 0.85, 0.98), and no significant benefit was observed for nebivolol (C07AB12), enalapril and diuretics (C09BA02) and losartan and diuretics (C09DA01) (Table 4). Nominally significant treatment effect modification was detected for age (interaction p-value 0.042), with the estimated treatment effects being stronger in patients aged 70 years or younger, aHR 1.20 (95% CI; 1.13, 1.28) than in relatively older patients, aHR 1.10 (95% CI; 1.04, 1.17), and for history of CVDD (interaction p < 0.001) (Table 4).

**Table 4:** Antihypertensive drugs: pooled IPTW-adjusted hazard ratios from subgroup analyses.

Subgroup	N branded	N generics	Mortality HR (95%CI)	p-value for interaction*	MACCE HR (95%CI)	p-value for interaction*
Females	232,229	304,174	1.17 (1.06, 1.28)	0.3075	1.14 (1.07, 1.22)	0.6376
Males	195,412	254,334	1.14 (1.04, 1.24)		1.13 (1.07, 1.19)	
Age < = 70 years	263,733	371,355	1.24 (1.11, 1.38)	0.0019	1.20 (1.13, 1.28)	<0.0001
Age > 70 years	163,908	187,153	1.13 (1.04, 1.22)		1.10 (1.04, 1.17)	
No history of CVDD	110,087	183,383	1.47 (1.31, 1.64)	<0.0001	1.34 (1.22, 1.47)	<0.0001
History of CVDD	317,554	375,125	1.10 (1.01, 1.20)		1.09 (1.03, 1.16)	
No diabetes	373,288	494,452	1.17 (1.07, 1.28)	0.0006	1.15 (1.08, 1.23)	0.0003
Oral DM therapy but no insulin use	46,134	56,091	1.10 (0.99, 1.22)		1.08 (1.01, 1.15)	
Insulin use	8,219	7,965	1.06 (0.96, 1.18)		1.08 (0.99, 1.18)	
Time-dependent effect: <= 6 months	427,641	558,508	1.12 (1.01, 1.24)	0.0876	1.28 (1.03, 1.58)	0.0111
>6 months	352,719	479,187	1.16 (1.06, 1.27)		1.13 (1.07, 1.20)	

*p-value for interaction of a variable with treatment, i.e., for testing the null hypothesis that HR is equal in the subgroups.

### Anti-hypertensives: treatment discontinuation

In 26.7% of all index prescriptions of branded medicines and also in 26.7% of all index prescriptions of generic medicines, no refill was observed within the first six months. However, after IPTW adjustment, the adjusted relative risk of treatment discontinuation was 1.23 (95% CI; 1.05, 1.44) in patients originally receiving branded medicines than in patients receiving generic medicines. In the landmark analysis including only patients who survived and were observed for at least six months and who were still using the originally prescribed medication, the aHR for mortality was very similar to the main analysis, aHR = 1.17 (95% CI; 1.03, 1.34), and not significantly different from the aHR computed in patients who discontinued treatment within six months from index prescription, aHR = 1.12 (95% CI; 1.04, 1.21) (p for interaction = 0.558). In the landmark analysis that included treatment discontinuation up to six months as a covariate, the aHR was virtually unchanged, aHR = 1.16 (95% CI; 1.04, 1.26).

### Lipid-lowering drugs: primary time-to-event outcomes

Patients using branded or generic lipid-lowering drugs experienced 24.4 (95% CI; 23.0, 25.9) and 16.0 (95% CI; 15.0, 17.2) deaths per 1000 patient-years, respectively. The IPTW-adjusted incidence rates per 1000 patient-years were 20.8 (95% CI; 19.9, 21.8) for branded drugs and 17.8 (95% CI; 16.9, 18.6) for generics. Cumulative five year survival rates were 86.6% (95% CI; 82.8%, 90.6%) and 91.1% (95% CI; 89.9%, 92.4%) in these two groups, and corresponded to adjusted survival rates of 89.0% (95% CI; 87.4%, 90.6%) and 90.6% (95% CI; 89.5%, 91.8%), respectively (Fig. 3).

The unadjusted pooled hazard ratio for mortality was 1.69 (95% CI; 1.09, 2.63), which was no longer significant after IPTW weighting, 1.13 (95% CI; 0.86, 1.47). For both individual substances, results suggested a lower hazard for generic medicines, however, results did not reach statistical significance for simvastatin (C10AA01). There was no significant effect modification by history of CVDD (p = 0.07) or time period (p = 0.35).

Branded and generic lipid-lowering drug users exhibited incidence rates for MACCE of 59.7 (95% CI; 57.4, 62.1) and 40.9 (95% CI; 39.1, 42.7) events per 1000 patient-years, respectively, which changed to 53.1 (95% CI; 51.6, 54.1) and 44.4 (95% CI; 43.0, 45.3) after IPTW adjustment. The pooled IPTW-adjusted hazard ratio was 1.20 (95% CI; 1.05, 1.38), and was more pronounced and significant for fluvastatin (C10AA04), while being smaller and non-significant in simvastatin (C10AA01). In subgroup analyses, we again observed a larger treatment effect estimate in patients with no previous history of CVDD, pooled aHR = 1.60 (95% CI; 1.19, 2.14), compared to patients with CVDD history, pooled aHR = 1.16 (95% CI; 1.03, 1.32), but interaction analyses failed to reach statistical significance (p = 0.052, Table 5). Similarly, there was no clear evidence for a time-dependent treatment effect (aHR for first six months, 1.37; aHR after six months, 1.10; interaction p = 0.151).

**Table 5:** Lipid-lowering drugs: pooled IPTW-adjusted hazard ratios from subgroup analyses.

Subgroup	N branded	N generics	Mortality HR (95%CI)	p-value for interaction*	MACCE HR (95%CI)	p-value for interaction*
Females	10,900	13,945	1.09 (0.79, 1.50)	0.3072	1.19 (1.04, 1.366)	0.3533
Males	10,765	11,749	1.18 (0.95, 1.47)		1.24 (1.07, 1.44)	
Age <= 70 years	15,381	18,658	1.07 (0.68, 1.69)	0.3721	1.18 (0.92, 1.51)	0.5031
Age > 70 years	6,284	7,036	1.18 (0.97, 1.43)		1.23 (1.08, 1.40)	
No history of CVDD	4,798	7,237	1.64 (1.14, 2.37)	<0.0001	1.60 (1.19, 2.14)	<0.0001
History of CVDD	16,867	18,457	1.08 (0.81, 1.43)		1.16 (1.03, 1.32)	
No diabetes	18,512	22,351	1.21 (1.01, 1.44)	0.2248	1.26 (1.14, 1.40)	0.0046
Oral DM therapy but no insulin use	2,727	2,977	0.85 (0.40, 1.81)		1.08 (0.86, 1.34)	
Insulin use	426	366	0.97 (0.36, 2.56)		0.76 (0.19, 3.06)	
Time-dependent effect: <= 6 months	21,665	25,694	1.32 (0.99, 1.77)	0.0294	1.37 (1.16, 1.62)	0.0009
> 6 months	19,055	22,857	1.08 (0.80, 1.45)		1.10 (0.86 1.41)	

*p-value for interaction of a variable with treatment, i.e., for testing the null hypothesis that HR is equal in the subgroups.

### Lipid-lowering drugs: treatment discontinuation

Treatment discontinuation rates were significantly higher in branded medicines with simvastatin (C10AA01), 43.4% vs. 27.6%, adjusted relative risk 1.80 (95% CI; 1.63, 1.99). However, discontinuation rates were virtually equal for fluvastatin (C10AA04), 31.5% vs. 32.2%, adjusted relative risk 1.03 (95% CI; 0.99, 1.08). A pooled relative risk estimate of 1.36 (95% CI; 0.79, 2.36) resulted for lipid-lowering drugs. Pooled adjusted hazard ratios for mortality from landmark analyses conditional on treatment continuation or discontinuation at six months were similar (interaction p-value = 0.75). Furthermore, including treatment discontinuation status at six month as covariate in a landmark analysis did not lead to a significant change in the overall result, aHR = 1.08 (95% CI; 0.80, 1.45).

### Hypoglycemic drugs: primary time-to-event outcomes

Incidence rates of mortality in patients using branded or generic hypoglycemic drugs were 55.5 (95% CI; 54.5, 56.6) and 29.8 (95% CI; 29.0, 30.6) events per 1000 patient-years, respectively. The corresponding IPTW-adjusted numbers were 45.9 (95% CI; 45.5, 46.9) and 40.3 (95% CI; 39.6, 40.9). Cumulative five-year survival rates were 75.1% (95% CI; 74.1%, 76.2%) and 85.0% (84.1%, 85.9%), respectively, and corresponded to IPTW adjusted survival rates of 77.4% (95% CI; 76.6%, 78.3%) and 81.9% (95% CI; 81.2%, 82.6%) (Fig. 3). The crude pooled hazard ratio for mortality of 1.43 (95% CI; 1.37, 1.49) reduced after IPTW adjustment to 1.09 (95% CI; 0.93, 1.28) (Table 2). A significantly lower mortality hazard for generics was observed for metformin (A10BA02) only, with aHR 1.21 (95% CI; 1.15, 1.27) (Table 3). Interaction tests did not reveal any significant differences between subgroups, nor between time periods (Table 6).

**Table 6:** Hypoglycemic drugs: pooled IPTW-adjusted hazard ratios from subgroup analyses.

Subgroup	N branded	N generics	Mortality HR (95%CI)	p-value for interaction*	MACCE HR (95%CI)	p-value for interaction*
Females	50,026	50,147	1.06 (0.85, 1,31)	0.3072	1.14 (1.03, 1.25)	0.2597
Males	51,019	49,846	1.12 (1.00, 1.25)		1.11 (1.06, 1.16)	
Age <= 70 years	63,719	70,352	1.19 (1.11,1.29)	0.0075	1.16 (1.11, 1.22)	0.0008
Age > 70 years	37,326	29,641	1.04 (0.84, 1.29)		1.07 (0.97, 1.18)	
No history of CVDD	15,754	21,629	1.06 (0.78, 1.43)	0.5352	1.04 (0.77, 1.40)	0.2720
History of CVDD	85,291	78,364	1.11 (0.96, 1.28)		1.12 (1.06, 1.19)	
No diabetes	61,401	78,420	1.14 (1.00, 1.30)	0.0475	1.15 (1.11, 1.19)	0.0027
Oral DM therapy but no insulin use	36,445	19,061	1.07 (0.93, 1.23)		1.09 (1.02, 1.16)	
Insulin use	3,199	2,512	0.97 (0.69, 1.38)		1.09 (0.84, 1.40)	
Time-dependent effect: <= 6 months	101,045	99,993	1.16 (1.06, 1.26)	0.0269	1.08 (1.02, 1.15)	0.3153
> 6 months	85,409	84,899	1.03 (0.82, 1.29)		1.11 (1.00, 1.24)	

*p-value for interaction of a variable with treatment, i.e., for testing the null hypothesis that HR is equal in the subgroups.

The incidence rates of MACCE for branded and generic hypoglycemic drug users were 88.5 (95% CI; 87.2, 89.9) and 54.2 (95% CI; 53.1, 55.3) events per 1000 patient-years, respectively. After IPTW adjustment, the corresponding MACCE incidence rate were 76.0 (95% CI; 75.1, 76.7) and 66.8 (95% CI; 66.0, 67.4). The IPTW-adjusted hazard ratio of MACCE confirmed a small but significant difference in favor of generics, aHR = 1.11 (95% CI; 1.03, 1.20) (Table 2), which was also seen in separate analyses of metformin (A10BA02) and gliclazide (A10BB09), while no difference was found for repaglinide (A10BX02) (Table 3). IPTW-adjusted hazard ratios in subgroups were very similar, and no differences in treatment effect could be confirmed by interaction tests. The aHR of MACCE did not change over time (Table 6).

### Hypoglycemic drugs: treatment discontinuation

Hypoglycemic treatment discontinuation rates at six months were 26.2% in branded users and 30.0% in generics users, and after IPTW-adjustments, there was no difference in the risk for discontinuation, with adjusted relative risk 1.02 (95% CI; 0.97, 1.07). In landmark analyses including only patients on treatment at six months, there was a clear benefit for metformin (A10BA02) generics users with respect to mortality, aHR = 1.27 (95% CI; 1.19, 1.35), but overall, the results pointed towards equivalence but with a wide confidence interval, pooled aHR = 1.01 (95% CI; 0.77, 1.34). In patients discontinuing their initial treatment, there was evidence for a small overall difference favoring generics, pooled aHR = 1.13 (95% CI; 1.04, 1.22). If the landmark analyses included treatment discontinuation as covariate, the pooled aHR was unchanged compared to the landmark analysis without further adjustment for treatment discontinuation, pooled aHR = 1.03 (95% CI; 0.83, 1.29).

## Discussion

We compared death and the incidence of MACCE for 17 branded versus generic versions of several medications commonly prescribed for chronic metabolic illnesses (hypertension or heart failure, hyperlipidemia, diabetes mellitus) within a national dataset representing nearly all insured persons in Austria from 2007 to 2012. Drawing from national hospitalization and pharmaceutical prescription fill data, we found a small but clear advantage for generic drugs over their branded counterparts for most of the studied substances. This generic advantage was robust across various levels of covariate adjustment, in landmark analyses considering drug discontinuation, and among sub-analyses based on age, sex, and previous disease status.

Among the studied patients, users of branded drugs generally appeared sicker than generic drug users. As shown in Table 1, branded drug patients had higher rates of recent hospitalizations, longer hospitalizations (and hospitals were more likely to initiate therapy with originator products), higher medication use, higher rates of previous MACCE events, and higher rates of copayment waivers suggesting lower socioeconomic status. As a result, unadjusted rates of mortality and MACCE favored generic drugs across all three drug categories. However, despite overall good covariate balancing achieved by IPTW weighting, the associations favoring generics were considerably attenuated but not eliminated after inverse probability of treatment weighting. Thus, residual confounding by unmeasured characteristics remains a possibility. Our sensitivity analyses supplied E-values between 1.4 and 1.69 for the point estimates. For comparison, the ratios of unadjusted and fully adjusted hazard ratios could be interpreted as the amount of bias removed by the considered covariates and ranged from 1.22 to 1.52. We find it unlikely that there is additional unmeasured confounding as strong as or even stronger than all measured covariates considered simultaneously.

Nevertheless, we believe disease severity is a possible source of unmeasured confounding. Because our data only included hospital discharge diagnoses instead of comprehensive medical records data with diagnoses from outpatient health care encounters, we were unable to identify patients with prior disease with high sensitivity. This would explain the large difference in adjusted hazard ratios among patients with and without prior CVDD for hypertension and hyperlipidemia drugs. The subgroups with prior CVDD more accurately reflect a sicker patient pool and have hazard ratios closer to 1, whereas the subgroups without prior CVDD may include patients with relatively more severe disease who were given branded medications. This subgroup difference based on CVDD disappeared when considering the diabetes drugs, and instead it is prior diabetes status that yielded a small but significant difference in subgroup analysis.

Prescribing doctor characteristics have been shown to affect medication prescription preferences, including generic substitution^30^. Physician skepticism about generic medication has been associated with age^31^, and pharmaceutical marketing^32^, and these trends may extend to Austrian physicians. In Austria, where generic substitution at pharmacies is generally prohibited by law, generic prescription lies entirely with the doctor. Unfortunately, we did not have detailed information on prescribing physicians other than specialty, and therefore could not extensively adjust for physician characteristics.

Two studied drugs had a maximum covariate standardized mean difference greater than 0.1 after IPTW weighting. These were bisoprolol (C07AB07) and repaglinide (A10BX02). Bisoprolol was associated with a higher copayment waive rate, 0.314 vs 0.265 among generic users, and perhaps lower socioeconomic status among generic users of bisoprolol contributes its being one of only two antihypertensive substances with significant branded drug advantage for mortality and MACCE outcomes. Repaglinide was associated with higher rates of dilated cardiomyopathy (as indicated by past discharge diagnoses) among generic users (0.011 vs 0.002). It was also associated with lower rates of mortality and MACCE among branded users, although this difference is not significant given the small sample size of repaglinide. These observations indicate that the observed outcomes are appreciably affected by imbalance among important covariates. For the other drugs that have maximum SMD smaller than 0.1, there may still be enough residual covariate imbalance after IPTW weighting to impact results. Perhaps other propensity score estimation methods than the high-dimensional propensity score may produce better covariate balance, such as including all covariates via lasso^33^ or machine learning algorithms^34^.

Medication adherence has previously been identified as a potential cause for the differences between generic and branded drug users, with the observation that the more expensive branded drugs engender lower drug adherence and therefore worse clinical outcomes^15,16,35^. However, other than for simvastatin (C10AA01), for which we observed higher branded discontinuation rates, we did not find significant differences in discontinuation among the studied drugs. As branded simvastatin was predominantly reimbursed in the 20 mg strength at the time of the study, some patients may have been switched to the generic 40 mg form for convenience, as this can be split, providing a longer duration of therapy per pack (and copayment). Landmark analyses using 6-month drug discontinuation also did not produce any significant differences. In Austria, copayment increased from 4.70€ in 2007 to 5.15€ in 2012. Since medication copayments are not different between branded and generic drugs and are generally low^3^, we did not expect drug discontinuation to be a prominent concern, as opposed to populations in systems with higher copayments or those with greater copayments for branded medications.

Our studied drugs are not representative of the narrow therapeutic index drugs that pose particular problems regarding generic substitution. Instead, we have studied common drugs for chronic diseases that could generate the greatest economic savings upon switching to generic formulations. Other studies focusing on similar therapeutic targets find nonsignificant differences between generic and branded drugs. A meta-analysis by Manzoli *et al*.^12^ of small randomized studies for cardiovascular drugs found no difference between generic and branded drugs for both soft and hard clinical outcomes. Randomized studies for statins also did not identify any differences in blood cholesterol levels between generic and branded users^36,37^. Ahrens *et al*.^38^ studied metoprolol in an observational study and found higher unadjusted cardiovascular event rates among generic users that disappeared upon confounder adjustment. Corrao *et al*.^14^ studied simvastatin in an observational study and found similar discontinuation rates and CV outcomes between generic and branded patients. By contrast, our results favored generic drugs both before and after adjustment. As discussed, this may be due to unmeasured confounding by indication with Austrian health providers, especially doctors in hospitals and specialists, perhaps preferring branded formulations for sicker patients, Although there is no biologically plausible rationale for this strategy, the economic incentives for choosing a particular brand or generic in the hospital setting in Austria can be different from those in the outpatient setting, because the systems of drug acquisition differ markedly in the two sectors^39^.

While the limitations of our study include its observational nature, which may lead to residual confounding from unobserved characteristics, there are also several strengths. These include a large study population that is nationally representative and conducted in a country with widely available access to healthcare, thus minimizing adherence differences and the impact of socioeconomic status as confounders. We used state-of-the-art propensity score methods to achieve good balance in observed covariates between compared groups, and we provide robust results with multiple subgroup and E-value sensitivity analyses. Future research would benefit from more detailed outpatient data to better characterize the patients’ health status, and more detailed prescriber characteristics, which could be achieved by linking additional data sources.

We conclude from this comprehensive study of almost all insured individuals in Austria that use of generic medications associated with similar or even slightly lower rates of mortality or nonfatal cardiovascular events. While there remains the potential for residual confounding by indication, our findings support the safety of policies towards greater use of generic substitute medications relative to their branded, and usually more expensive, versions.

## Supplementary information

Supplementary material.
