# Supplementary material for: Comparative effectiveness of branded vs. generic versions of antihypertensive, lipid-lowering and hypoglycemic substances: a population-wide cohort study
Source: Sci Rep. 2020 Apr 6;10:5964. doi: 10.1038/s41598-020-62318-y (PMC7136234; doi:10.1038/s41598-020-62318-y)
Supplement: Supplementary file 1 — Supplementary material. [file 41598_2020_62318_MOESM1_ESM.pdf]

Supplementary material:

Comparative effectiveness of branded vs. generic versions of antihypertensive, lipid-lowering and hypoglycemic substances: a population-wide cohort study

Yuxi Tian[1], Berthold Reichardt[2], Daniela Dunkler[3], Milan Hronsky[3], Wolfgang C. Winkelmayer[4], Anna Bucsics[5], Susanne Strohmaier[3], Georg Heinze[3,*]

[1] Department of Biomathematics, University of California Los Angeles, Los Angeles, CA, USA

[2] Sickness Fund of the Province of Burgenland, Eisenstadt, Austria

[3] Center for Medical Statistics, Informatics, and Intelligent Systems, Section for Clinical Biometrics, Medical University of Vienna, Vienna, Austria

[4] Section of Nephrology, Baylor College of Medicine, Houston, TX, USA

[5] MoCA, Mechanism of Coordinated Access to Orphan Medicinal Products, Austria, Vienna

* Corresponding author.

Address for correspondence:

Medical University of Vienna

Center for Medical Statistics, Informatics and Intelligent Systems

Section for Clinical Biometrics

Spitalgasse 23

1090 Vienna

Austria

E-mail: georg.heinze@meduniwien.ac.at

Supplementary table 1: Performance of high-dimensional propensity models

Indication<br>Substance	ATC code	Weighted maximum* SMD**	C-index***
Antihypertensive drugs			
Metropolol	C07AB02	7.00%	0.703
Bisoprolol	C07AB07	12.35%	0.763
Nebivolol	C07AB12	6.35%	0.727
Carvedilol	C07AG02	4.19%	0.766
Amlodipine	C08CA01	0.61%	0.760
Enalapril	C09AA02	8.64%	0.639
Lisinopril	C09AA03	2.80%	0.739
Ramipril	C09AA05	0.85%	0.837
Enalapril and diuretics	C09BA02	6.17%	0.681
Lisinopril and diuretics	C09BA03	5.17%	0.748
Ramipril and diuretics	C09BA05	5.82%	0.762
Losartan and diuretics	C09DA01	8.66%	0.854
Lipid-lowering drugs			
Simvastatin	C10AA01	8.15%	0.714
Fluvastatin	C10AA04	6.11%	0.714
Hypoglycemic drugs			
Metformin	A10BA02	2.48%	0.670
Gliclazide	A10BB09	7.21%	0.700
Repaglinide	A10BX02	17.33%	0.758

*maximum over the 200 top-prioritized covariates identified by the HDPS algorithm
**SMD: absolute standardized mean difference. The weighted standardized mean difference measures the imbalance in characteristics between patients treated with branded and generic drugs. After inverse probability of treatment weighting to increase balance, a value lower than 10% is usually considered as indicating adequate balance.
***C-index: concordance index of the high-dimensional propensity score model. The c-index measures the discriminative ability of the propensity model. It expresses the probability that in a randomly selected pair of patients, one treated with a branded, one with a generic drug, the patient treated with the branded drug has a higher estimated probability to receive a branded drug. A value of 0.5 indicates random treatment assignment, while a value of 1 indicates perfect predictability of treatment assignment from the patient characteristics.
